# Clinical Application of Surgical Guides in MARPE: Observational Research

**DOI:** 10.3390/jcm15155787

**Published:** 2026-07-24

**Authors:** Eugen-Silviu Bud, Mariana Pacurar, Ana-Petra Lazar, Bucur Sorana-Maria, Anamaria Bud, Luminta Lazar, Andrei Cosmin Nenec, Alexandru Vlasa

**Affiliations:** 1 Department of Orthodontics, Faculty of Dental Medicine, George Emil Palade University of Medicine, Pharmacy, Science, and Technology of Târgu Mureș, 38 Ghe. Marinescu Street, 540139 Târgu Mureș, Romania; eugen.bud@umfst.ro (E.-S.B.); mariana.pacurar@umfst.ro (M.P.); 2 Department of Oral Rehabilitation, Faculty of Dental Medicine, George Emil Palade University of Medicine, Pharmacy, Science, and Technology of Târgu Mureș, 38 Ghe. Marinescu Street, 540139 Târgu Mureș, Romania; 3 Faculty of Medicine, Dimitrie Cantemir University of Târgu Mureș, 3-5 Bodoni Sandor Street, 540545 Târgu Mureș, Romania; 4 Department of Pedodontics, Faculty of Dental Medicine, George Emil Palade University of Medicine, Pharmacy, Science, and Technology of Târgu Mureș, 38 Ghe. Marinescu Street, 540139 Târgu Mureș, Romania; anamaria.bud@umfst.ro; 5 Department of Periodontology, Faculty of Dental Medicine, George Emil Palade University of Medicine, Pharmacy, Science, and Technology of Târgu Mureș, 38 Ghe. Marinescu Street, 540139 Târgu Mureș, Romania; luminita.lazar@umfst.ro (L.L.); alexandru.vlasa@umfst.ro (A.V.); 6 Stomatology and Maxilo-Facial Surgery Department, Targu Mures Emergency County Hospital, 540136 Târgu Mureș, Romania; andrei_nenec@outlook.com

**Keywords:** palatal expansion, surgical guides, virtual planning, palatal suture

## Abstract

**Background/Objectives:** Miniscrew-assisted rapid palatal expansion (MARPE) is an effective treatment for maxillary transverse deficiency in skeletally mature patients. However, resistance of the midpalatal suture may limit treatment success. Surgical corticopunctures have been proposed to facilitate suture opening, while digital planning and 3D-printed surgical guides may improve procedural accuracy and safety. This study aims to evaluate the clinical effectiveness and safety of MARPE combined with surgically guided midpalatal corticopunctures in adult patients. **Methods:** A retrospective observational study was conducted on 15 adult patients (at least 20 years old) presenting with maxillary transverse deficiency and midpalatal suture maturation stages D or E. All patients underwent corticopuncture-assisted MARPE using patient-specific 3D-printed surgical guides designed through CBCT-based virtual planning. Treatment success was assessed by postoperative cone-beam computed tomography (CBCT), which evaluated midpalatal suture opening and transverse expansion. Clinical records were reviewed for complications, mini-implant stability, and postoperative outcomes. **Results**: Successful opening of the midpalatal suture was achieved in 13 of 15 patients, corresponding to a success rate of 86.7%. Among successful cases, the mean suture expansion measured on CBCT was 3.6 ± 0.9 mm. The customized surgical guides demonstrated adequate intraoral fit and enabled accurate execution of the planned corticopunctures in all cases. No intraoperative guide-related complications were reported. Postoperative healing was uneventful, with only mild and transient discomfort observed. No infections, excessive bleeding, significant soft-tissue injuries, damage to adjacent anatomical structures, or adverse events related to the corticopuncture procedure were recorded. Mini-implant stability was maintained in the majority of patients throughout the expansion phase. **Conclusions**: Within the limitations of this retrospective single-arm study, corticopuncture-assisted MARPE performed using customized 3D-printed surgical guides was feasible and associated with a high rate of successful midpalatal suture opening and few complications. The technique demonstrated a high rate of suture opening, clinically significant skeletal expansion, and a low incidence of complications. Digital planning and guided execution may enhance treatment precision and improve clinical outcomes in MARPE procedures.

## 1. Introduction

Maxillary transverse deficiency, also known as maxillary constriction, is a transverse deficiency of the upper jaw where the jaw is too narrow. It causes a high, narrow palate, dental crowding, and crossbites. It is often linked to mouth breathing, affecting 21% of children and 10% of adults, according to various studies [1,2,3].

Failure to correct maxillary transverse deficiency may contribute to the worsening of sagittal and vertical malocclusions, compromised masticatory efficiency, and a greater likelihood of obstructive sleep apnea syndrome (OSAS). All of which may negatively affect patients’ general health and quality of life [4]. Orthopedic widening of the maxilla is therefore a key treatment objective. In growing patients, rapid palatal expansion (RPE) is widely used to achieve transverse correction, particularly during the mixed dentition period [5]. Its effectiveness decreases after skeletal maturation because the midpalatal suture becomes increasingly interdigitated and resistant to separation. For this reason, conventional RPE often produces an insufficient skeletal response in adults, and other expansion techniques should be considered [6].

In adult patients, skeletal widening of the maxilla is commonly achieved through miniscrew-assisted rapid palatal expansion (MARPE) or surgically assisted rapid palatal expansion (SARPE). Despite its less invasive nature, MARPE may be associated with adverse effects, including soft-tissue irritation and inflammation around the appliance, local infection, and periodontal damage. It can also lead to loss of miniscrew stability caused by prolonged pressure on the palatal mucosa [7].

To reduce some of these adverse effects of MARPE, it is important to use surgical guides through correct placement of mini-implants in orthodontics [8]. These guides make the work of doctors much more predictable and bring the benefits of reducing the risk of damage to neighboring teeth. It achieves it by creating a predictable path for insertion of mini-implants while maintaining a predetermined insertion length and angulation [9].

Surgical guides use biocompatible materials and high-accuracy 3D images are needed for their planning [10]. These images are obtained using cone-beam computed tomography (CBCT) that shows the dental structures in detail. The tomographic images obtained are superimposed on scans of the dental arches, allowing a high-precision design (CAD) of future surgical guides [11].

The time required to create surgical guides has been greatly reduced by using a digital workflow that includes CBCT, intraoral scanners, CAD/CAM systems and 3D printers [12].

Miniscrew-assisted rapid palatal expansion (MARPE) has emerged as an innovative approach to address maxillary transverse discrepancies in adults—a group often resistant to traditional methods due to skeletal maturity. As maxillary expansion is critical in orthodontic treatments, its successful execution depends significantly on precise surgical techniques, particularly the accurate placement of mini-implants [13]. The clinical complications associated with the placement of these miniscrews can significantly impact patient outcomes, including failure and discomfort [14].

Recent advancements in digital technology, particularly in three-dimensional (3D) printing, have revolutionized surgical planning by allowing the creation of tailored surgical guides. Such guides facilitate precise miniscrew placement, thereby enhancing the efficiency and effectiveness of the MARPE procedure [15]. The role of these surgical guides in improving the predictability of surgical outcomes while minimizing procedural complications is an area of growing interest among orthodontic professionals [1].

In the context of MARPE, surgical guides allow orthodontists to visualize and achieve the intended anatomical targets, aiding in overcoming the anatomical barriers present in mature patients. Proper planning, using cone-beam computed tomography (CBCT) images, enhances the customization of these guides by aligning them with the unique anatomical architecture of each patient, thus allowing for an optimized surgical experience [16].

Despite the promising potential of utilizing surgical guides in MARPE, systematic reviews and comparative studies on their efficacy remain limited. The existing literature highlights the need for comprehensive observational studies to assess the robustness of this technology in real-world settings, focusing on the outcomes of patients treated with the assistance of surgical guides as opposed to those receiving standard treatments without them [17,18,19].

The success of MARPE is influenced by accurate mini-implant positioning, which can be enhanced using surgical guides. Treatment predictability is also strongly affected by the degree of ossification of the midpalatal suture and by other anatomical areas of resistance. To achieve skeletal expansion with MARPE, the applied force must be sufficient to overcome resistance within the midfacial complex, including the zygomatic buttresses, pterygomaxillary junctions, piriform aperture pillars, and the midpalatal suture.

Several approaches have been proposed to facilitate maxillary expansion in adult patients. These include CBCT-based, digitally designed surgical guides for three-dimensional guided midpalatal piezocorticotomy-assisted MARPE, as well as minimally invasive surgically assisted miniscrew-anchored rapid palatal expansion (sa-MARPE), which avoids conventional horizontal maxillary osteotomies. The latter technique involves three small mucosal incisions and limited piezosurgical osteotomies targeting the midpalatal suture, canine pillars, and pterygomaxillary junctions. By preserving mucosal integrity and vascular supply, this approach may reduce postoperative morbidity and promote faster recovery [20].

A major limitation of these adjunctive surgical techniques is patient reluctance, as they are still perceived as invasive, although to a much lesser extent than SARPE. In our clinical practice, corticopunctures have long been performed at the level of the midpalatal suture to weaken suture resistance and facilitate expansion. However, we sought to make this procedure more predictable by using digital planning to avoid relevant anatomical structures and to achieve controlled bone removal along the midpalatal suture. Therefore, the present study aimed to evaluate the treatment efficacy of MARPE combined with surgically guided micropunctures of the palatal suture.

### Objectives

The primary objectives of this observational study are to assess the following:

1. The success rate of suture expansion associated with the use of surgical guides for micropunctures in MARPE procedures.
2. The occurrence of complications arising from expansion facilitated by these micropunctures surgical guides.

## 2. Materials and Methods

The study protocol was reviewed and approved by the Ethics Committee of Algocalm (approval no. 92/15.01.2026).

### 2.1. Study Design

An observational retrospective study was conducted to evaluate cases of MARPE and corticopunctures performed using 3D-printed surgical guides over a designated period at an orthodontic clinic. Patients were included consecutively using the following criteria:

### 2.2. Selection Criteria

Inclusion criteria:

- Patients aged 20 years or older undergoing MARPE, with midpalatal suture matu- ration stage D or E (established by Angelieri et al.) [21];
- Availability of preoperative imaging (CBCT) for surplanning via the guides.

### 2.3. Data Collection

Data were retrieved from electronic health records:

- Patient demographics (age, sex).
- Preoperative and postoperative CBCT measurements regarding the width of the maxillary suture were performed at the inter-incisal line Cemental–Enamel Junction. This anatomical landmark was choose because it is stable and reproductible during tooth movement.
- Documented complications (e.g., miniscrew failures, infections).

**Figure 1:**
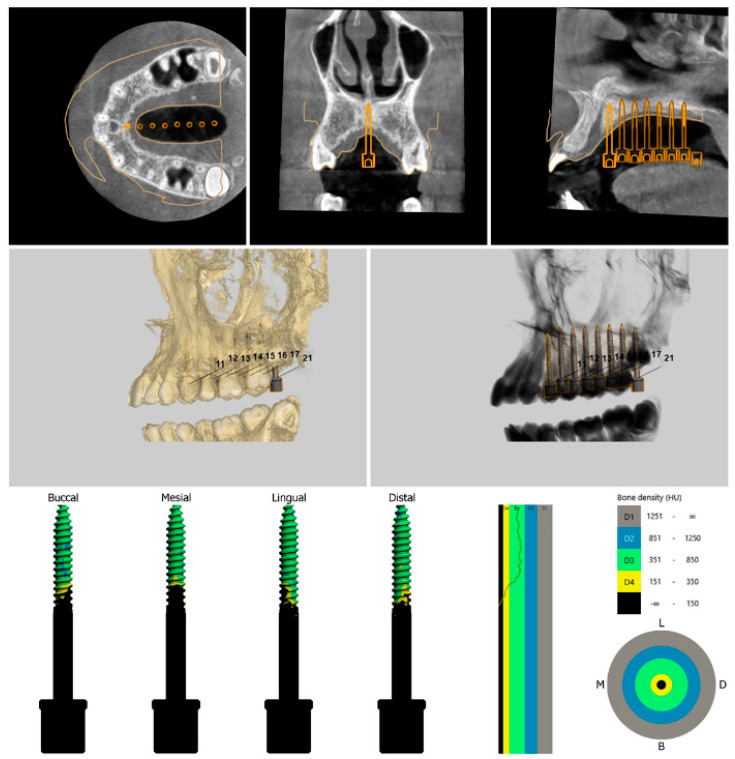
Virtual planning used to create the surgical guide necessary to perform microperforations of the median palatal suture.

**Figure 2:**
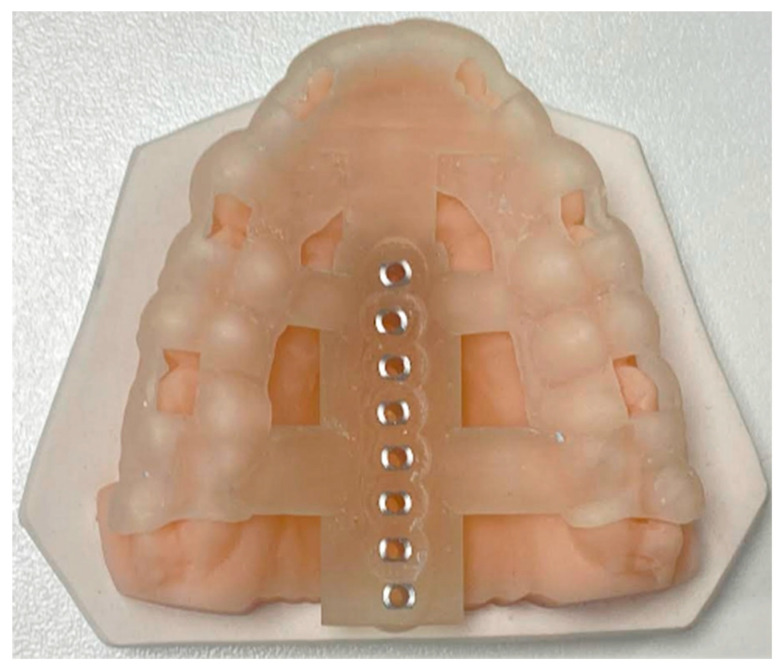
Surgical guide manufactured.

The surgical guide was designed using 3Shape Implant Studio software, version 2026.1 (3Shape A/S, Copenhagen, Denmark). The surgical guide (Figure 1 and Figure 2) was fabricated for a Cowellmedi anchor sleeve (code KLSAS01), and only the anchor drill with a diameter of 1.3 mm (code KLSAD13) was used. Because the upper part of the anchor sleeve had a diameter of 3.5 mm, a maximum of only 7–10 perforations could be performed. The corticopuncture procedure was carried out under greater palatine nerve block anesthesia and local infiltration. Topical Lidocaine™ Septodont (Creteil, France) 2% spray was applied for 1 min, followed by buccal infiltration of the hard palate with a solution of articaine hydrochloride and epinephrine 1:100,000 (ARTICAINE™ Septodont, Creteil, France), administered using a 0.30 × 38 mm gingival needle (Heraeus™, Hanau, Germany). After the procedure, patients were prescribed analgesic medication for pain relief and instructed to use a 0.12% chlorhexidine mouthrinse for 7 days. All patients followed the same activation protocol for MSE II™: minimum 4~6 turns/day (0.53~0.80 mm/day) until a gap was observed between the central incisors, indicating success in separating the midpalatal suture.

**Figure 3:**
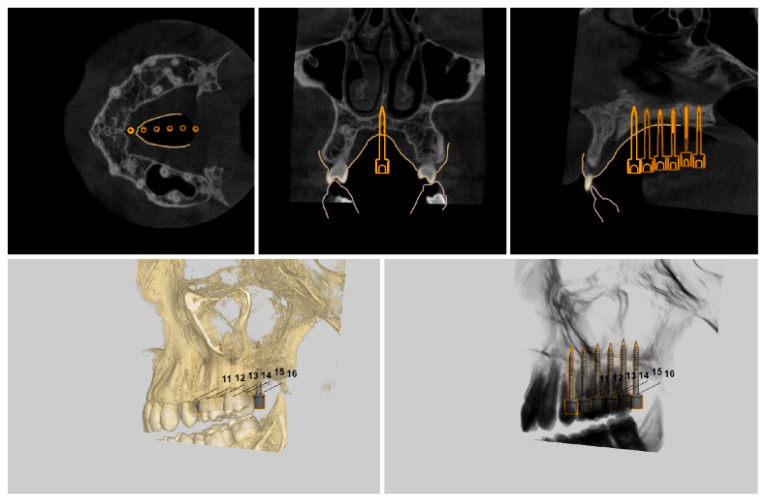
A case in which the first perforation is performed more distally due to the ramifications of the incisive canal.

**Figure 4:**
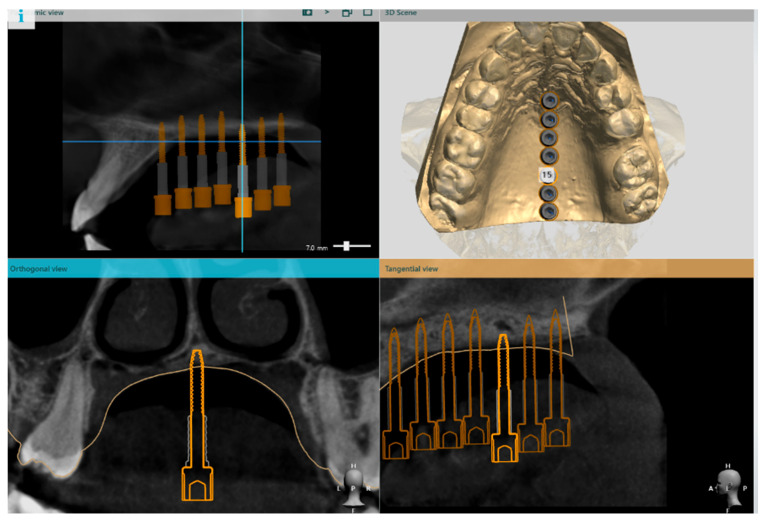
Sixth perforation took into account the existence of a formation at the median suture level (possibly a blood vessel) without damaging the respective formation.

**Figure 5:**
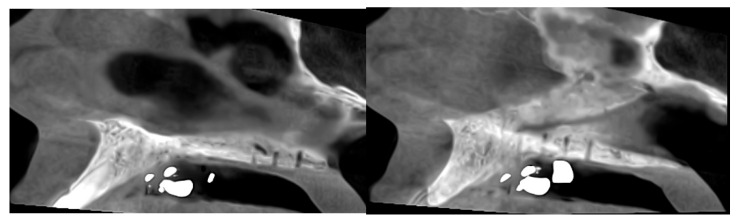
Virtual planning of the guide-assisted perforations ensured accuracy in both the transverse and vertical planes, allowing controlled weakening of the midpalatal suture resistance.

**Figure 6:**
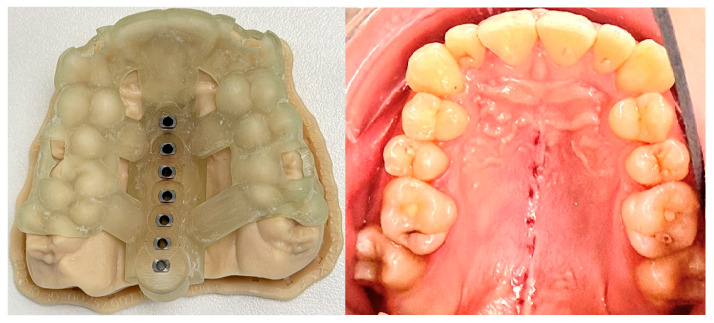
Perforations performed based on the surgical guide.

Due to anatomical variations, virtual planning was individualized according to each patient’s morphology, as illustrated in Figure 3, Figure 4, Figure 5 and Figure 6.

### 2.4. Statistical Analysis

Statistical analysis was limited to descriptive statistics. Continuous variables are presented as mean ± standard deviation, whereas categorical variables are expressed as frequencies and percentages. Owing to the observational single-arm design, no comparative statistical analyses were performed. Because of the limited sample size (*n* = 15), statistical power was limited and the findings should be interpreted cautiously. The results should therefore be considered preliminary and require confirmation in larger prospective studies.

## 3. Results

A total of 15 adult patients with midpalatal suture maturation stages D or E, according to the classification proposed by Angelieri et al. [21], met the inclusion criteria and were included in the study. The study group consisted of nine males and six females, mean age 23.4 years old. All patients underwent corticopuncture-assisted microimplant-assisted rapid palatal expansion (MARPE) using a digitally planned, patient-specific 3D-printed surgical guide.

Successful opening of the midpalatal suture was achieved in 13 of the 15 treated patients, corresponding to an overall success rate of 86.7% (Table 1). In these patients postoperative CBCT evaluation confirmed radiographic separation of the midpalatal suture, indicating successful skeletal expansion. Two patients did not exhibit radiographic evidence of sufficient suture opening and were therefore classified as treatment failures.

Quantitative CBCT analysis showed a mean midpalatal suture expansion of 3.6 ± 0.9 mm among the successfully treated cases. This amount of expansion was considered clinically relevant and indicates that the combined corticopuncture and MARPE protocol could produce skeletal changes in patients with advanced stages of suture maturation. Postoperative imaging demonstrated a clear increase in transverse maxillary dimensions, with separation occurring along the midpalatal suture.

The use of the 3D-printed surgical guides enabled accurate and reproducible positioning of the corticopunctures in relation to the planned anatomical landmarks. In all cases, the guides exhibited adequate intraoral fit and stability during the procedure, allowing the perforations to be performed according to the virtual treatment plan. No intraoperative difficulties were encountered with surgical guide positioning or access to the surgical site.

Clinical follow-up revealed a favorable postoperative course in most patients. Mild postoperative discomfort and localized tenderness in the palatal region were commonly reported during the first few days after the procedure. The discomfort was resolved spontaneously, in some cases with the help of prescribed analgesic medication. Healing of the palatal mucosa was uneventful in all patients.

No major complications were observed throughout the treatment period. Specifically, no cases of postoperative infection, excessive bleeding, significant soft-tissue injury, or adverse reactions associated with the surgical guide were recorded. Likewise, no damage to adjacent anatomical structures was identified clinically or radiographically. The guided corticopuncture protocol was well tolerated by the patients and did not result in any unexpected adverse events.

Most MARPE devices maintained mini-implant stability throughout the expansion phase. The high success rate observed in this study suggests that precise digital planning and guide-assisted execution may support predictable force distribution and effective orthopedic expansion in adult patients.

Overall, the results demonstrated that corticopuncture-assisted MARPE performed with the aid of a customized 3D-printed surgical guide achieved successful maxillary expansion in most patients included in the study. The technique was associated with a high rate of midpalatal suture opening, clinically meaningful skeletal expansion, and a low incidence of complications.

Adverse events included postoperative bleeding requiring intervention, infection requiring antibiotics, guide instability, soft-tissue injury, injury to adjacent anatomical structures, mini-implant instability and delayed wound healing. These were actively evaluated during scheduled follow-up visits through standardized clinical examinations and review of patient records.

## 4. Discussion

Surgical Precision and Planning: Studies show that the use of surgical guides during surgical procedures allows for improved precision. The ability to visualize the target anatomy via advanced imaging techniques ensures that orthodontists can place miniscrews in optimal locations and properly reduce the strength of the midpalatal suture through micropunctures, lowering complications and leading to higher success rates [22,23,24,25,26]. Research confirms that inaccuracies during placement can result in miniscrew failure and suboptimal maxillary expansion. The overall goal in orthodontics is to ensure that treatment is as minimally invasive as possible, while also effectively achieving the desired changes [27].

Improvement in Patient Outcomes: Enhanced clinical outcomes connected with the use of surgical guides indicate a relationship between surgical precision and patient satisfaction. The data collected may support claims from the existing literature that assert heightened satisfaction levels when physicians implement advanced planning and technology into their practice. This leads to offering patients tailored treatment regimens alongside reduced chair time during visits [22,28]. The removal of guesswork through a guided process is likely to also foster a better rapport between the clinician and the patient as trust increases.

Technological Integration and Digital Workflow: The intersection of digital imaging and 3D printing holds transformative potential for the orthodontic field. Enhanced protocols that utilize preoperative diagnostics alongside guided surgeries will likely continue to shape orthodontic practices in the future. Advanced planning workflows provide a fit that ensures the most efficient use of clinical resources and improves the patient experience in the process [7].

Comparative Analysis of MARPE Approaches: In the context of studies comparing MARPE with conventional expansion methods, the use of surgical guides may enhance both skeletal and dentoalveolar outcomes after expansion while improving treatment stability. Available comparative data suggests that guide-assisted corticopuncture combined with MARPE may be associated with fewer adverse effects than more invasive surgical approaches commonly used in adult patients, potentially reducing postoperative recovery time and supporting broader acceptance of minimally invasive treatment protocols.

Surgical Precision in Corticopunction and Piezotomy: Surgical guides play a critical role during corticopuncture and piezotomy procedures designed to disrupt the midpalatal suture. Enhanced precision offered by these guides helps to ensure accurate perforation of the suture and bolster the effectiveness of the subsequent expansion process [13] (Koval et al., 2025). By employing 3D-printed surgical guides tailored to each patient’s anatomy, orthodontists can effectively navigate areas of resistance within the suture and avoid complications typically associated with less precise methods [13] (Koval, 2025). This precision allows for the timely activation of palatal expanders and reduces the likelihood of patients experiencing side effects related to improper screw placements.

Accuracy and Outcomes: Several studies indicate that using surgical guides contributes to enhanced success rates and suture expansion post-MARPE. Koval et al. highlighted through their case report that 3D-guided piezocorticotomy assisted MARPE leads to predictable skeletal expansion while attenuating unilateral expansion asymmetries [13]. Ensuring proper suture retention and mitigating the complications linked to conventional methods—where inaccuracies can result in extended healing times or malocclusions—offers an exciting consideration for integrating surgical guides into everyday practice [29].

Technological Integration: The landscape of orthodontics is evolving with the advent of digital technologies. Utilizing 3D imaging to design surgical plans enables orthodontists to customize their approaches according to individual patient needs. The use of surgical guides enhances the reliability of micropunctures and miniscrew placement, directly influencing the mechanical efficacy of the MARPE procedure in inducing suture expansion [30,31]. In addition, advances in digital workflows support clinician training, improve procedural standardization, and may facilitate wider implementation of MARPE techniques in clinical practice [32].

Comparative Analysis with Traditional Methods: Observational studies comparing MARPE, facilitated by surgical guides, with conventional RME or SARPE demonstrate the benefits of guided procedures in reducing the potential for complications and enhancing clinical outcomes [13]. A systematic review indicated that patients receiving MARPE experienced more favorable treatment outcomes than those utilizing conventional techniques, with fewer side effects and greater overall satisfaction [33]. Collectively, these findings underscore the advancements surgical guides bring to the field, enriching both the orthodontist’s understanding and the patients’ experiences.

Suzuki performed corticopuncture with a good outcome in a 35-year-old patient with a previous failed MARPE treatment by inserting and removing a 9 mm titanium alloy miniscrew (5 mm double thread, 4 mm neck of length and 1.8 mm diameter). The distance between perforations was 2 mm. It is important to note that corticopunctures were only 5 mm depth in the midpalatal suture. In the anterior part of the palate perforations were done carefully to avoid the nasopalatine canal [34].

Having performed both conventional midpalatal micropunctures and those guided by surgical guides, we found the guided approach to be more predictable, based on the comparison of pre- and postoperative CBCT scans.

Limitations and Future Directions: This study acknowledges potential limitations, including the challenge of obtaining a sufficiently large and diverse patient population within a specific timeframe. Future studies should consider a multicenter approach to corroborate the findings and better establish the efficacy of surgical guides for MARPE across a broader demographic [13,15]. As additional research sheds light on the application and efficacy of surgical guides in piezotomy and other surgical interventions, we can anticipate advancements in orthodontic practices that improve successful patient outcomes and reduce treatment times.

Limitations and Need for Further Research: While this study aims to provide robust data challenging existing limitations pertaining to surgical guides, it is essential to note possible constraints such as sample size or demographic homogeneity. A longitudinal approach across multiple centers may yield a more generalizable understanding of the impacts of surgical guides in MARPE. Furthermore, exploring long-term outcomes associated with the esthetic and functional changes following surgical guide interventions will prove insightful.

Alternatives if Micropunctures Do Not Work: Balercia describes a minimally invasive, surgically assisted MARPE protocol designed to overcome these limitations while avoiding complete Le Fort I osteotomies. The technique involves three small mucosal incisions and limited piezosurgical osteotomies targeting the midpalatal suture, canine pillars, and pterygomaxillary junctions. By preserving mucosal integrity and vascular supply, this approach reduces postoperative morbidity and enhances recovery [20]. Recent evidence also highlights the need for standardized outcome reporting in studies evaluating MARPE. In an umbrella review, Ventura et al. [33] synthesized the available systematic reviews and concluded that MARPE is an effective treatment modality for achieving skeletal maxillary expansion, while reducing some of the dentoalveolar side effects associated with conventional rapid maxillary expansion. However, the authors also emphasized that the overall certainty of the evidence remains limited due to methodological heterogeneity, variable study designs, and the predominance of observational investigations. These findings underscore the importance of conducting well-designed prospective and randomized clinical studies with standardized protocols and long-term follow-up to establish stronger evidence regarding the efficacy and predictability of guide-assisted MARPE techniques. Furthermore, beyond transverse skeletal correction, increasing attention has been directed toward the functional effects of MARPE. A recent systematic review and meta-analysis by Li et al. [35] demonstrated that microimplant-assisted rapid palatal expansion is associated with significant increases in upper airway volume, particularly within the nasal cavity and nasopharyngeal regions, suggesting that the benefits of MARPE may extend beyond orthodontic correction to improvements in respiratory function. The continued evolution of digital technologies is expected to further improve the precision and predictability of surgically assisted MARPE. Three-dimensional planning, computer-guided piezocorticotomy, and additive manufacturing of patient-specific surgical guides have the potential to optimize the positioning of osteotomies and miniscrews while minimizing surgical trauma and reducing operator-dependent variability. A recent case report by Koval et al. [36] demonstrated the successful integration of three-dimensional guided piezocorticotomy-assisted MARPE with directly printed aligners for the management of complex maxillary asymmetry, illustrating the potential of comprehensive digital workflows to improve treatment accuracy and interdisciplinary coordination. Future investigations should therefore incorporate functional outcomes, including airway changes, breathing quality, and patient-reported quality-of-life measures, alongside conventional skeletal and dental assessments.

This study has several limitations. First, its retrospective single-arm design without a control group precludes comparisons with conventional MARPE or free-hand corticopuncture techniques and does not permit causal inference regarding the contribution of the surgical guide to treatment success. Second, the relatively small sample size limits statistical power and reduces the generalizability of the findings. Third, the retrospective design is inherently susceptible to selection bias, although all eligible patients treated during the study period were included. Finally, only immediate post-expansion outcomes were evaluated. Long-term stability, relapse, and patient-reported outcomes were not assessed and should be investigated in future prospective controlled studies.

## 5. Conclusions

The findings of this observational study will illuminate the role of surgical guides within the context of MARPE procedures, reinforcing the essential advantages provided by 3D planning technologies. By emphasizing treatment precision, improved clinician–patient communication, and efficient resource utilization, this study supports the continued evolution of orthodontic standards of care toward individualized, patient-centered approaches that optimize treatment outcomes. Controlled prospective studies are required to determine whether this protocol offers advantages over conventional approaches. Long-term skeletal stability remains unknown and should be investigated in future prospective studies with extended follow-up.

## Figures and Tables

**Table 1:** Clinical and radiographic outcomes of corticopuncture-assisted MARPE using 3D-printed surgical guides (*N* = 15).

Variable	Result
Total number of patients (*N*)	15
Patients with successful midpalatal suture opening	13
Patients without successful suture opening	2(1 case converted to SARPE, one case-repeated MARPE)
Success rate	86.7%
Mean suture expansion (successful cases)	3.6 ± 0.9 mm
Type of imaging used for assessment	CBCT
Intraoperative guide-related complications	None reported
Postoperative infections	None reported
Significant soft-tissue injuries	None reported
Excessive bleeding	None reported
Adverse events related to corticopuncture	None reported
Mini-implant stability during expansion	Stable in the majority of cases
Postoperative discomfort	Mild and transient
Healing of palatal tissues	Uneventful in all patients

## Data Availability

Supporting reported results can be checked with the corresponding authors.
